# Editorial: Endometrial health and disease: from molecular insights to clinical advances

**DOI:** 10.3389/fvets.2026.1860973

**Published:** 2026-05-26

**Authors:** Ana Amaral, Katarzyna Piotrowska-Tomala, Pawel Kordowitzki

**Affiliations:** 1 CIISA—Centre for Interdisciplinary Research in Animal Health, Faculty of Veterinary Medicine, University of Lisbon, Lisbon, Portugal; 2 Associate Laboratory for Animal and Veterinary Sciences (AL4AnimalS), Lisbon, Portugal; 3 Comprehensive Health Research Centre (CHRC), Évora, Portugal; 4 InLife—Institute of Animal Reproduction and Food Research, Polish Academy of Science, Olsztyn, Poland; 5 Department of Basic and Preclinical Sciences, Institute for Veterinary Medicine, Nicolaus Copernicus University, Torun, Poland; 6 Department of Gynecology, European Competence Center for Ovarian Cancer, Charité Medical University, Berlin, Germany

**Keywords:** endometritis, endometrium, microbiome, translational models, uterine inflammation

The endometrium is a highly dynamic and functionally complex tissue that plays a central role in reproductive success across species. Its cyclical remodeling, driven by tightly regulated hormonal cues, is essential for embryo implantation, pregnancy maintenance, and overall uterine health. Disruptions in these finely tuned processes are associated with a wide spectrum of reproductive disorders, including infertility, early embryonic loss, and chronic uterine disease, with significant implications for both animal health and reproductive efficiency.

Recent advances in molecular biology, omics technologies, and reproductive biotechnology have substantially expanded our understanding of endometrial physiology and pathology. These developments have enabled the identification of key regulatory pathways involved in immune modulation, tissue remodeling, and host–microbe interactions, as well as their dysregulation in disease states. In parallel, there is growing recognition that endometrial health is shaped not only by local factors but also by systemic conditions, highlighting the need for integrative and translational approaches.

**Figure 1:**
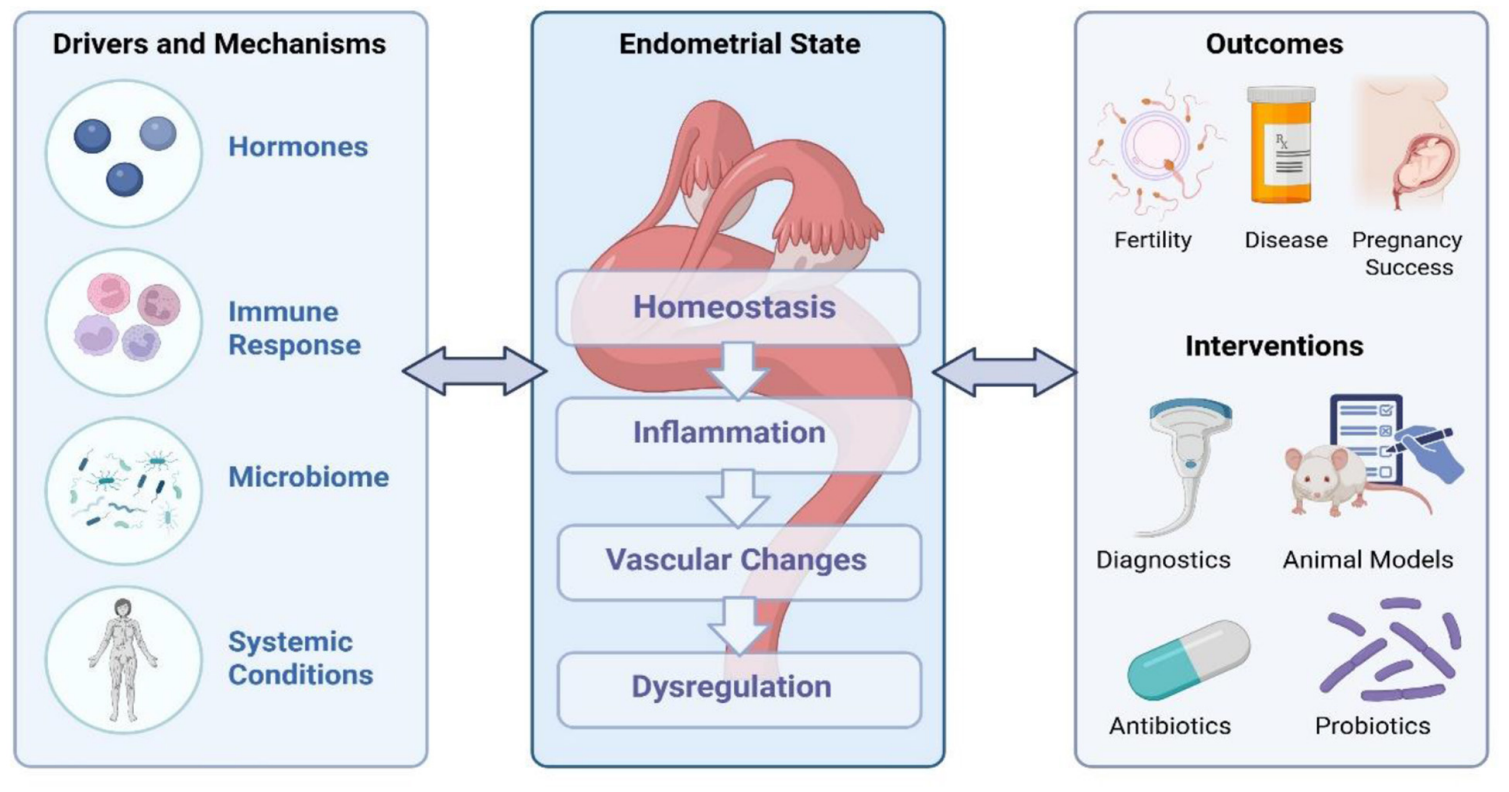
Overview of the Research Topic “*Endometrial health and disease: from molecular insights to clinical advances*” (created in BioRender).

This Research Topic brings together six articles that collectively provide a multidisciplinary perspective on endometrial health and disease across animal species, encompassing diagnostic innovations, mechanistic insights, experimental models, and emerging therapeutic strategies (Figure 1).

A central theme emerging from this collection is the complexity and clinical relevance of uterine inflammation, particularly in its subclinical or dysregulated forms. One study, by Sahu et al., demonstrates that subclinical endometritis in dairy cows is associated with distinct alterations in uterine hemodynamics, characterized by increased blood flow and reduced vascular resistance, as assessed by transrectal Doppler ultrasonography. These findings highlight the potential of non-invasive imaging techniques to detect otherwise undiagnosed inflammatory conditions, offering promising avenues for improving reproductive management under field conditions.

Complementing this, the Howard et al., study explores the impact of pituitary pars intermedia dysfunction, a systemic endocrinopathy, on the equine reproductive tract. The authors show that affected mares exhibit increased expression of pro-inflammatory cytokines, particularly interleukin-8, alongside leukocyte infiltration within the endometrium and follicular environment. These findings underscore the concept that systemic disease can directly influence uterine immune status, positioning the endometrium as a responsive interface between systemic physiology and reproductive function.

Beyond the characterization of inflammation, this Research Topic also highlights the importance of its resolution. In mares susceptible to persistent breeding-induced endometritis, supplementation with resveratrol was shown to modulate the uterine inflammatory response, reducing neutrophil infiltration and altering cytokine profiles, as described by Denison et al. Rather than simply suppressing inflammation, such approaches may promote a more controlled and timely resolution of the post-breeding inflammatory cascade, which is critical for maintaining fertility. This distinction between persistent and properly resolved inflammation represents an important conceptual shift with therapeutic implications.

The need for innovative and sustainable treatment strategies is further emphasized by studies addressing antimicrobial use in cows with endometritis. Pas et al., *in vitro* study of the evaluation of alternative antimicrobial compounds revealed that while several agents exhibit antibacterial activity against common uterine pathogens, their cytotoxic effects on endometrial cells vary considerably. These findings highlight the importance of balancing antimicrobial efficacy with tissue safety, particularly in the context of increasing efforts to reduce antibiotic use under the One Health framework.

In line with this, another study made by Liu et al., investigates the therapeutic potential of a probiotic strain, *Lactiplantibacillus plantarum*, in a murine model of *Escherichia coli*-induced endometritis. The results demonstrate not only a reduction in inflammatory markers and tissue damage but also beneficial modulation of the uterine microbiota. Such approaches reflect a paradigm shift toward microbiome-targeted therapies, which may offer safe and effective alternatives to conventional antimicrobial treatments.

Importantly, the translational relevance of these findings is reinforced by Zhao et al., comprehensive review examining the pig as a model for gynecological diseases. Due to its anatomical and physiological similarities to humans, the porcine model provides a valuable platform for studying endometrial disorders, evaluating therapeutic interventions, and bridging the gap between veterinary and human medicine. This perspective emphasizes that advances in animal reproductive research have far-reaching implications beyond species-specific applications, contributing to broader biomedical knowledge.

Collectively, the articles in this Research Topic illustrate the multifaceted nature of endometrial health and disease, integrating molecular, cellular, and clinical dimensions. They highlight the importance of early and accurate diagnosis, the intricate interplay between systemic and local factors, and the need for innovative therapeutic strategies that address both inflammation and infection while preserving tissue integrity.

Future research in this field will benefit from increasingly integrative approaches that combine advanced diagnostics, mechanistic insights, and translational models. In particular, further exploration of immune regulation, host–microbiome interactions, and fibrosis-related processes may provide new opportunities for improving reproductive outcomes across species.

In conclusion, this Research Topic provides a comprehensive overview of current advances in endometrial biology and pathology, offering valuable insights for both veterinary practice and translational research. By bridging fundamental science and clinical application, it contributes to a deeper understanding of reproductive health and supports the development of more effective and sustainable strategies for managing endometrial disease.

